# Knowledge, Attitude, and Practice on Atrial Fibrillation and Atrial Fibrillation Screening in High‐Risk Population: A Cross‐Sectional Study

**DOI:** 10.1002/agm2.70030

**Published:** 2025-07-15

**Authors:** Zhong Yi, Xinxin Yan, Shaozhi Xi, Lijuan Zhu, Rui Liu, Jing Li, Songwei Ru, Xiu Zheng, Jingxuan Qiu, Chunqi Wang, Na Guo, Yimin Wang, Dayong Gao

**Affiliations:** ^1^ Department of Geriatrics Aerospace Center Hospital Beijing China; ^2^ PLA General Hospital Department of Cardiology Beijing China; ^3^ Internal Medicine Clinic Aerospace Center Hospital Beijing China; ^4^ Department of Medical Records Management Aerospace Center Hospital Beijing China; ^5^ Yongding Road Community Health Service Center Beijing China

**Keywords:** atrial fibrillation, atrial fibrillation screening, attitude, high‐risk elderly population, knowledge, practice

## Abstract

**Objectives:**

Atrial fibrillation (AF) is a common clinical arrhythmia. Failure to identify AF leaves patients at a considerably higher risk of disability and death, making its early screening and prevention extremely important. This study aimed to investigate knowledge, attitude, and practice (KAP) towards AF prevention and screening among the high‐risk population.

**Methods:**

Individuals at a high risk of AF were required to fill out questionnaires between August 2022 and October 2022.

**Results:**

A total of 863 participants aged 66.54 ± 11.00 years were enrolled. The median scores of knowledge, attitude, and practice were 9 (81.8% of maximum), 37 (64.9% of maximum), and 21.56 (86.2% of maximum), respectively. Only knowledge (OR = 1.159, 95% confidence interval (CI): 1.045–1.286, *p* = 0.005) was independently associated with better practice, while attitude (OR = 0.861, 95% CI: 0.817–0.908, *p* < 0.001), reported poor sleep quality (OR = 0.529, 95% CI: 0.259–1.0788, *p* = 0.010), fair (OR = 0.234, 95% CI: 0.111–0.491, *p* < 0.001) or fluctuating (OR = 0.321, 95% CI: 0.103–0.996, *p* = 0.049) emotional state, and acquiring the questionnaire through social media (OR = 0.419, 95% CI: 0.268–0.653, *p* < 0.001) were independently associated with worse practice.

**Conclusions:**

Participats from high‐risk AF population in this study demonstrated acceptable practice towards AF screening, mostly moderated by knowledge and attitude. Some gaps in knowledge could be improved by targeted health education, especially important in vulnerable subpopulations.

## Graphical abstract

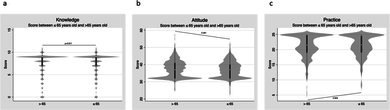

**Abbreviations**
AF: Atrial fibrillation
APHRS: Asia Pacific Heart Rhythm Society
ECG: electrocardiogram
EHRA: European Heart Rhythm Association
HRS: Heart Rhythm Society
KAP: knowledge, attitude, and practice
SOLAECE: Sociedad Latinoamericana de Estimulación CardíacayCardíaca y Electrofisiología

## 1 Background

Atrial fibrillation (AF) is one of the most common arrhythmias in the world, with prevalence increasing gradually at older age [1, 2]. The presence of AF significantly increases the risk of ischemic cerebral stroke and other cardiovascular complications, elevating disability and mortality rates [3, 4]. In addition, the incidence of asymptomatic AF is very high, especially in the elderly [5]. Thus, reducing the burden of AF and embolism has become one of the challenges for modern medicine. Considering that the diagnosis of AF depends on an electrocardiogram (ECG), asymptomatic AF is generally difficult to discover [6]. Screening of high‐risk populations can improve the detection rate of AF, which is helpful for early detection, diagnosis, and treatment [7].

A variety of screening options were proposed for early AF detection, but theoretical research and practical experience have indicated that individual practice depends on the knowledge and attitude of each patient [8]. Only when the acquired basic knowledge is truly absorbed can healthy practice be promoted [9]. Therefore, it is of great significance to evaluate the knowledge, attitudes, and practices (KAP) of AF and AF screening in high‐risk populations and explore their influencing factors so that targeted interventions can be implemented. A few previous KAP studies were mainly focused on the diagnosis and treatment of AF [10, 11, 12], while KAP towards screening is rarely discussed, especially among the elderly population who are harder to reach. Based on the above, this cross sectional survey was conducted to explore the KAP towards AF prevention (including oral anticoagulants, blood pressure and blood glucose monitoring, lifestyle changes) and AF screening (including different screening modalities) in high‐risk elderly participants.

## 2 Methods

### 2.1 Study Design and Participants

This cross sectional study was performed online between August 2022 and October 2022. Patients aged ≥ 65 years or with at least one of the following characteristics were included: hypertension, diabetes, coronary heart disease, chronic obstructive pulmonary disease, obstructive sleep apnea‐hypopnea syndrome, hyperthyroidism, hypothyroidism, history of AF in close family members, or heavy drinking (more than 5 drinks every day or 15 drinks per week). Exclusion criteria were the inability to fill out the online questionnaire. The study was approved by the Ethics Committee of the Aerospace Center Hospital (CR‐2021‐CHDRP (2020)‐002). Informed consent was obtained from all participants.

### 2.2 Procedures

A self‐administered questionnaire on AF prevention and screening was designed according to the European Heart Rhythm Association (EHRA) consensus document endorsed by the Heart Rhythm Society (HRS), Asia Pacific Heart Rhythm Society (APHRS), Sociedad Latinoamericana de Estimulación Cardíaca y Electrofisiología (SOLAECE) [13], 2016 ESC Guidelines for the management of AF developed in collaboration with EACTS [14], and 2021 Asia Pacific Heart Rhythm Society (APHRS) practice guidance on AF screening [15]. The questionnaire was reviewed by 3 experts (including 2 cardiovascular experts and 1 epidemiologist) and modified according to the suggestions. The final questionnaire consisted of the following 4 dimensions: (1) demographic characteristics of participants, including 18 items; (2) knowledge dimension, including 12 questions. The correct answer to each question was given 1 point, while every wrong answer or unclear answer was given 0 points. The total score of the knowledge dimension ranged from 0 to 12 points. (3) Attitude dimension included 12 questions and was evaluated with the 5‐level Likert scale; for questions signifying positive attitudes (A1, A2, A5, A10, A12), 5 points were allocated for the “strongly agree” and 1 point for “strongly disagree”. For questions signifying negative attitudes (A3, A4, A6, A7, A8, A9, and A11), 1 point was allocated for “strongly agree” and 5 points for “strongly disagree”. The total score for 12 questions ranged from 12 to 60 points. (4) Practice dimension included 5 questions (question P5 additionally devided into 8 subquestions) and was also evaluated using the 5‐level Likert scale. The questions were scored from 5 points for “always” to 1 point for “never”, and the total score of the dimension ranged from 5 to 25 points.

The final questionnaire had a Cronbach's α of 0.913 and Kaiser‐Meyer‐Olkin (KMO) of 0.923, indicating that the high internal consistency [16]. The investigators established the online questionnaire based on the Questionnaire Star platform (https://www.wjx.cn/), after which a Quick Response (QR) code was generated. Participants were recruited from the outpatient department and wards of the hospital, as well as social media. Participants scanned the QR code to complete the questionnaire survey. The investigators assessed the completeness, internal continuity, and rationality of all questionnaires; then the trained staff reviewed the data for errors and verified data by telephone or during the next visit.

### 2.3 Statistical Analysis

Stata 17.0 software (Stata Corporation, College Station, TX, USA) was used for the statistical analysis. Continuous data were expressed as mean ± standard deviation (SD) and compared by *t*‐test. The categorical data were presented as *n* (%) and compared with the chi‐squared test. The KAP scores were compared between subgroups of participants with different characteristics. The Practice scores were used as the dependent variables, after which the multivariate analysis was used to explore the association of demographic characteristics with KAP. The results were categorized using 70% of the Practice scores as the cutoff [17]. Variables with *p* < 0.05 in the univariate model were included in the multivariate analysis. All statistical analyses were two‐sided, and *p* < 0.05 was considered statistically significant.

## 3 Results

### 3.1 Demographic Characteristics

A total of 863 patients with a mean age of 66.54 ± 11.00 years were enrolled, including 270 males (31.29%) and 587 females (68.02%). The Body Mass Index (BMI) of participants ranged from 15 to 37.64 kg/m^2^, with an average of 23.53 kg/m^2^. The mean scores of knowledge, attitude, and practice were 9 (range from 0 to 11), 37 (range from 23 to 57), and 21.56 (range from 5 to 25), respectively.

The higher knowledge scores were found among urban residents (*p* < 0.001), those with higher educational levels (*p* = 0.012), professional and technical staff/employees (*p* < 0.001), higher income groups (*p* < 0.001), married individuals (*p* = 0.019), those with social medical insurance only (*p* = 0.017), nonsmokers (*p* = 0.009), nondrinkers, and current drinkers (as compared to previous drinkers, *p* = 0.039), a more stable emotional state (*p* < 0.001), and in those who received the questionnaire at the outpatient department or ward of the hospital (*p* < 0.001). In terms of attitude scores, the higher attitude scores were observed in female participants (*p* < 0.001), those who were unmarried (*p* = 0.010), social medical insurance and commercial medical insurance holders (*p* = 0.002), nonsmokers and previous smokers (*p* = 0.046), current drinkers (*p* = 0.037), in those who reported no history of AF (*p* = 0.001), fair sleep (*p* < 0.001), and a stable emotional state (*p* < 0.001), as well as in those who received the questionnaire at the outpatient department or ward of the hospital (*p* < 0.001). As for practice scores, the higher practice scores were found in urban residents (*p* = 0.008), professional and technical staff (*p* < 0.001), higher income groups (*p* < 0.001), married individuals (*p* = 0.001), social medical insurance and commercial medical insurance holders (*p* = 0.034), nonsmokers (*p* < 0.001), nondrinkers (*p* < 0.001), those who reported previous AF (*p* < 0.001), better sleep (*p* < 0.001), and a highly stable emotional state (*p* < 0.001) and in those who received the questionnaire at the outpatient department or ward of the hospital (*p* < 0.001) (Table 1).

**TABLE 1:** Baseline characteristics and comparison of knowledge, attitude, and practice scores between participants with different characteristics.

Variables	*n* = 863	Knowledge		Attitude		Practice	
		Score	*p*	Score	*p*	Score	*p*
Total score		9 (0, 11)		37 (23, 57)		21.56 (5, 25)	
Age	66.54 ± 11.00						
BMI	23.53 (15, 37.64)						
Sex			0.067		< 0.001		0.051
Male	270 (31.29)	9 (6, 9)		34 (32, 39)		22.05 (19, 25)	
Female	587 (68.02)	9 (7, 9)		38 (33, 41)		21.43 (19.06, 24.37)	
Missing	6 (0.70)	/		/		/	
Residence			< 0.001		0.056		0.008
Urban area	781 (90.50)	9 (7, 9)		37 (32, 41)		21.75 (19.25, 24.68)	
Nonurban area	82 (9.50)	7 (3, 9)		36 (32, 39)		20.15 (16.75, 24)	
Educational level			0.012		0.108		0.073
Primary school or lower	88 (10.20)	8 (5, 9)		36 (32, 39)		20.75 (18.15, 24.59)	
Junior middle school/senior middle school/technical secondary	259 (30.01)	9 (7, 9)		37 (32, 40)		21 (18.93, 24.31)	
Junior college/college	479 (55.50)	9 (7, 9)		37 (32, 41)		21.93 (19.30, 24.68)	
Postgraduate or higher	37 (4.29)	9 (7, 9)		35 (32, 39)		23 (20, 25)	
Occupation			< 0.001		0.245		< 0.001
Professional and technical staff	415 (47.97)	9 (8, 9)		37 (32, 41)		22.42 (19.93, 24.93)	
Employee	194 (22.48)	9 (6, 9)		36 (32, 39)		20.77 (18.66, 24.5)	
Others	253 (29.32)	8 (5, 9)		37 (33, 40)		20.73 (18.12, 24)	
Missing	2 (0.23)						
Income (yuan)			< 0.001		0.518		< 0.001
≤ 5000	175 (20.28)	8 (4, 9)		37 (34, 41)		20.46 (17.4, 23)	
5001–10,000	473 (54.81)	9 (7, 9)		37 (32, 41)		21.93 (19.31, 24.93)	
≥ 10,001	210 (24.33)	9 (7, 9)		37 (32, 40)		22.08 (19.87, 24.68)	
Missing	5 (0.58)						
Marital status			0.019		0.010		0.001
Unmarried	117 (13.56)	8 (5, 9)		38 (35, 41)		20.62 (18.14, 23.31)	
Married	746 (86.44)	9 (7, 9)		37 (32, 40)		21.79 (19.25, 24.81)	
Underlying disease*							
Diabetes	331 (38.35)	9 (8, 9)		32 (32, 37)		24.81 (21.5, 25)	
Hypertension	459 (53.19)	9 (7, 9)		35 (32, 40)		22.8 (19.66, 25)	
Renal disease	21 (2.43)	8 (7, 9)		32 (31, 37)		21.62 (20.5, 24.5)	
Coronary heart disease	141 (16.34)	9 (7, 9)		38 (35, 41)		20.93 (18.56, 23.06)	
Bradycardiac arrhythmia	115 (13.33)	9 (7, 9)		35 (32, 40)		22.42 (19.56, 25)	
Valvulopathy	14 (1.62)	8 (7, 9)		37 (33, 40)		20.87 (19.56, 24)	
Hyperthyroidism	11 (1.27)	9 (8, 9)		38 (32, 40)		21.46 (19.5, 24.87)	
Hypothyroidism	41 (4.75)	8 (8, 9)		39 (36, 41)		20.06 (18.06, 22.6)	
Respiratory disease	59 (6.84)	8 (7, 9)		37 (34, 41)		20.46 (18, 22.56)	
None	169 (19.58)	9 (5, 9)		39 (35, 42)		21 (18.81, 23.75)	
Others	122 (14.14)	8 (5, 9)		38 (35, 41)		20.27 (17.93, 22.68)	
Medical insurance			0.017		0.002		0.034
Social medical insurance only	796 (93.32)	9 (7, 9)		37 (32, 40)		21.75 (19.06, 24.81)	
Social medical insurance and commercial medical insurance	27 (3.17)	7 (5, 9)		40 (36, 44)		21.25 (18.62, 24.26)	
None	30 (3.52)	8 (4, 9)		39 (35, 41)		20.07 (17.81, 23.18)	
Smoking			0.009		0.046		< 0.001
Nonsmoker	723 (84.66)	9 (7, 9)		37 (32, 40)		22 (19.5, 24.87)	
Previous smoker	88 (10.30)	8 (5, 9)		37 (35, 41)		20.03 (17.31, 22.14)	
Current smoker	43 (5.04)	8 (5, 9)		35 (32, 38)		19.75 (16.43, 23.81)	
Alcohol drinking			0.039		0.037		< 0.001
Nondrinkers	647 (76.03)	9 (7, 9)		37 (32, 41)		22.12 (19.68, 24.87)	
Previous drinkers	114 (13.40)	8 (5, 9)		37 (32, 39)		20.86 (18, 23.87)	
Current drinkers	90 (10.58)	9 (6, 9)		38 (34, 41)		19.84 (17, 22)	
Family history of atrial fibrillation			0.002		0.325		0.092
Yes	114 (13.32)	9 (8, 9)		37 (32, 40)		22.25 (19.5, 25)	
No	742 (86.68)	9 (7, 9)		37 (32, 41)		21.43 (19, 24.5)	
History of atrial fibrillation			< 0.001		0.001		< 0.001
Previous atrial fibrillation	120 (14.04)	9 (8, 9)		34 (32, 39)		23 (19.5, 25)	
Current atrial fibrillation	54 (6.32)	9 (7, 9)		37 (32, 40)		21.11 (18.66, 24.93)	
None	502 (58.71)	9 (7, 9)		38 (32, 41)		22 (19.42, 24.62)	
Unclear	179 (20.94)	8 (4, 9)		37 (33, 40)		20.31 (18, 23.5)	
Sleep score			< 0.001		< 0.001		< 0.001
Very good	232 (26.95)	9 (9, 9)		32 (32, 33)		25 (24, 25)	
Good	222 (25.78)	9 (6, 9)		37.47 ± 4.37		20.87 (18.87, 23.5)	
Fair	273 (31.71)	8 (6, 9)		38.95 ± 4.54		20.18 (18.31, 22.64)	
Poor/very poor	134 (15.56)	8 (6, 9)		38.84 ± 4.05		20.62 (18, 22.5)	
Emotional state			< 0.001		< 0.001		< 0.001
Highly stable	265 (30.71)	9 (9, 9)		32 (32, 35)		24.93 (23.56, 25)	
Stable	301 (34.88)	9 (6, 9)		38.63 ± 4.37		20.73 (19, 23.06)	
Fair	239 (27.69)	8 (5, 9)		38.46 ± 4.51		20.12 (17.81, 22.31)	
Fluctuating	58 (6.72)	8 (7, 9)		37.67 ± 3.94		20.53 (17.93, 23)	
Questionnaire access			< 0.001		< 0.001		< 0.001
Hospital	414 (47.97)	9 (8, 9)		33 (32, 39)		24 (20.1, 25)	
Social Media	449 (52.03)	8 (6, 9)		38.50 ± 4.33		20.53 (18.14, 22.5)	

*Note*: *indicates that the question is a multiple‐choice question.

### 3.2 Distribution of Each Dimension

Table 2 shows the distribution and correct rate of questions in the knowledge dimension. It was found that some items had a higher correct rate, while other items had a very low correct rate, which should be greatly improved. Notably, the correct rate of “*The incidence of atrial fibrillation increases with age.”* (K3), “*Atrial fibrillation can elevate the risk of ischemic stroke and systemic circulation arterial embolism*.” (K5), and “*The screening of asymptomatic atrial fibrillation can only be done in hospital*.” (K12) was < 10%.

**TABLE 2:** Knowledge dimension.

Statements	*n* (%)	
	Correct	Wrong/Unclear
K1. Atrial fibrillation is one of the most common arrhythmias in clinical practices.	664 (76.94)	199 (23.06)
K2. Atrial fibrillation includes paroxysmal, persistent, and permanent atrial fibrillation.	603 (70.20)	256 (29.80)
K3. The incidence of atrial fibrillation increases with age.	51 (5.93)	809 (94.07)
K4. The major manifestation of atrial fibrillation includes palpitation, discomfort in the chest, shortness of breath, and fatigue.	686 (79.77)	174 (20.23)
K5. Atrial fibrillation can elevate the risk of ischemic stroke and systemic circulation arterial embolism.	11 (1.28)	850 (98.72)
K6. The three key measurements of preventing atrial fibrillation are to prevent stroke, control the ventricular rate, and control the cardiac rhythm.	659 (76.36)	204 (23.64)
K7. Modifying unhealthy lifestyles could reduce the risk of atrial fibrillation.	729 (84.97)	129 (15.03)
K8. Atrial fibrillation screening could help in the early detection, diagnosis, and treatment of atrial fibrillation.	788 (91.73)	71 (8.27)
K9. Managing hypertension, smoking, and alcohol drinking can reduce the risk of atrial fibrillation.	768 (89.30)	92 (10.70)
K10. Detecting and diagnosing asymptomatic atrial as early as possible is critical for oral anticoagulation therapy and stroke prevention.	712 (82.79)	148 (17.21)
K11. Screening is especially important for patients with asymptomatic atrial fibrillation.	768 (89.41)	91 (10.59)
K12. The screening of asymptomatic atrial fibrillation can only be done in the hospital.	29 (3.38)	830 (96.62)

**FIGURE 1:**
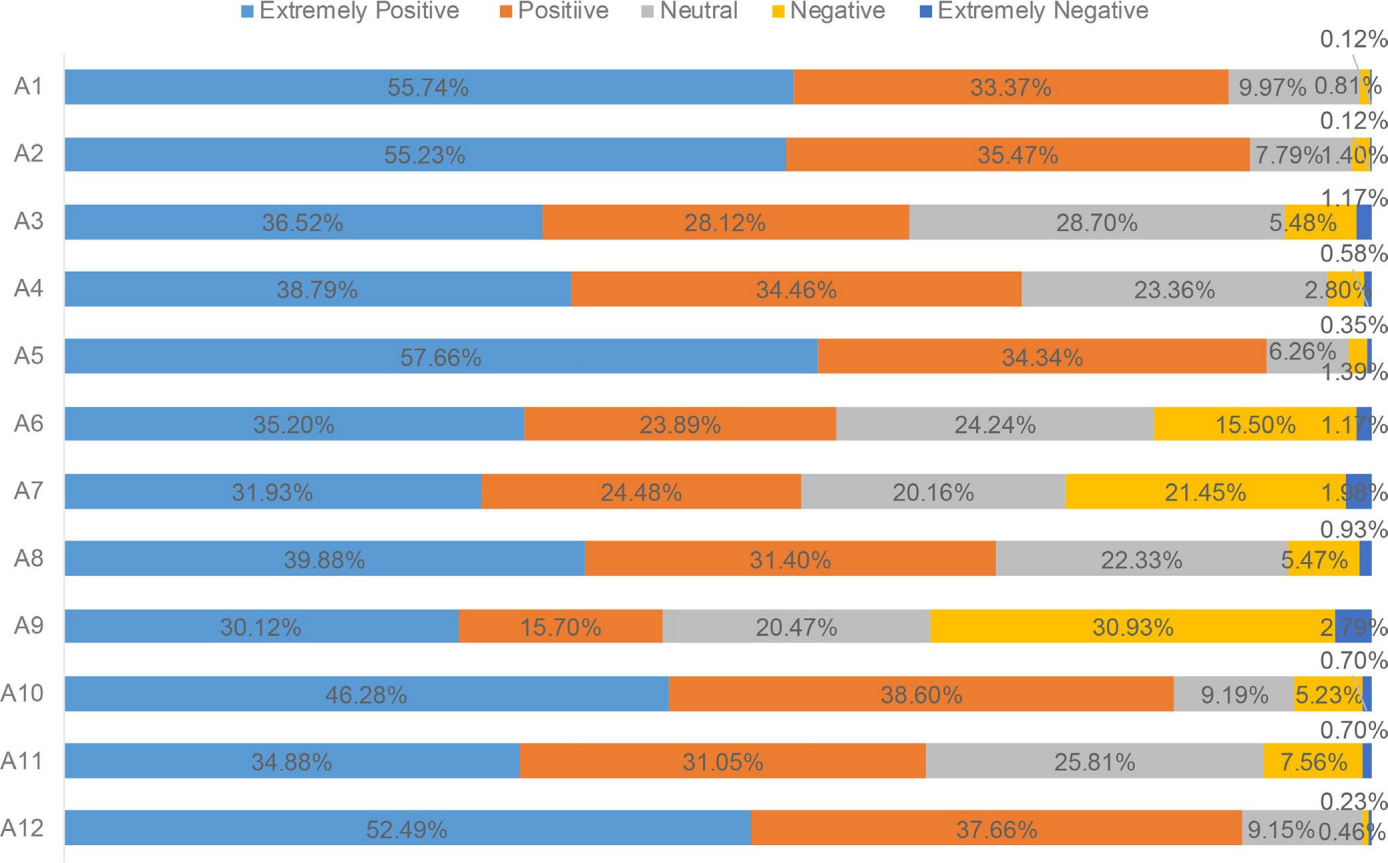
The score distribution of “attitude” dimension. (A1) I believe atrial fibrillation is a dangerous disease (P); (A2) I believe atrial fibrillation can reduce patients' quality of life (P); (A3) I think the precautions for taking anticoagulants are too complicated (N); (A4) I think anticoagulation therapy for atrial fibrillation takes too long (N); (A5) I can benefit from atrial fibrillation screening (P); (A6) I'm worried that atrial fibrillation screening will be too expensive (N); (A7) I am worried that atrial fibrillation screening will not be useful (N); (A8) I am terrified of having atrial fibrillation (N); (A9) I think a regular review of atrial fibrillation is a waste of time (N); (A10) I would be more than willing to do so if I could be screened at any time without going to the hospital (P); (A11) I would be very anxious if the screening result was positive (N); (A12) I would like to learn more about atrial fibrillation and atrial fibrillation screening (P).The score distribution of “attitude” dimension. (A1) I believe atrial fibrillation is a dangerous disease (P); (A2) I believe atrial fibrillation can reduce patients' quality of life (P); (A3) I think the precautions for taking anticoagulants are too complicated (N); (A4) I think anticoagulation therapy for atrial fibrillation takes too long (N); (A5) I can benefit from atrial fibrillation screening (P); (A6) I'm worried that atrial fibrillation screening will be too expensive (N); (A7) I am worried that atrial fibrillation screening will not be useful (N); (A8) I am terrified of having atrial fibrillation (N); (A9) I think a regular review of atrial fibrillation is a waste of time (N); (A10) I would be more than willing to do so if I could be screened at any time without going to the hospital (P); (A11) I would be very anxious if the screening result was positive (N); (A12) I would like to learn more about atrial fibrillation and atrial fibrillation screening (P).

The participants' attitudes towards AF prevention and screening are presented in Figure 1. Nearly 90% of the participants considered AF as a dangerous disease that reduces patients' quality of life (agree or completely agree); 60%–70% of the participants considered anticoagulation treatment for AF to be complex and prolonged therapy. Furthermore, the majority of the participants considered AF screening beneficial and would like to learn more about AF and AF screening. However, about half of the participants worried that AF screening was too expensive and insufficient to detect any problems. Additionally, 46% of the participants considered regular screening to be time‐consuming, and 85% hoped to have the possibility to undergo AF screening at any time without going to the hospital.

**FIGURE 2:**
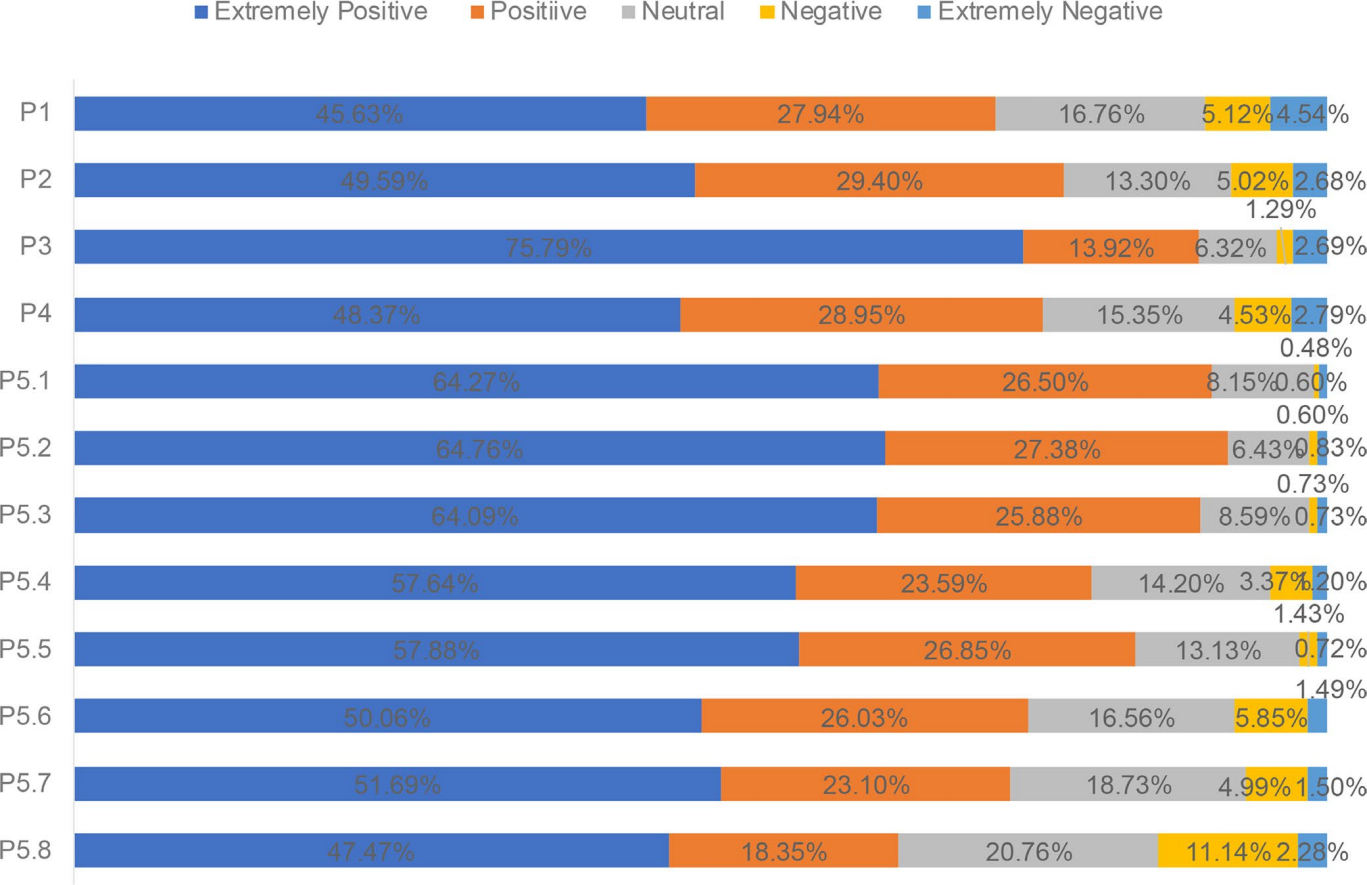
The score distribution of “practice” dimension. (P1) I will take oral anticoagulants if recommended by my doctor; (P2) I will closely monitor the blood pressure and blood glucose level to prevent the occurrence of atrial fibrillation; (P3) I can quit smoking and drinking alcohol to prevent the occurrence of atrial fibrillation; (P4) I will exercise with appropriate intensity to prevent the occurrence of atrial fibrillation; (P5) Are you willing to use the following AF screening modalities: P5.1 pulse palpation; P5.2 blood pressure (heart rate) measurement; P5.3 electrocardiogram (resting 12‐lead electrocardiogram); P5.4 dynamic electrocardiogram (a small box over the shoulder); P5.5 echocardiography; P5.6 smartphone + APP; P5.7 smartwatch/bracelet +APP; P5.8 wearable vest. The score distribution of “practice” dimension. (P1) I will take oral anticoagulants if recommended by my doctor; (P2) I will closely monitor the blood pressure and blood glucose level to prevent the occurrence of atrial fibrillation; (P3) I can quit smoking and drinking alcohol to prevent the occurrence of atrial fibrillation; (P4) I will exercise with appropriate intensity to prevent the occurrence of atrial fibrillation; (P5) Are you willing to use the following AF screening modalities: P5.1 pulse palpation; P5.2 blood pressure (heart rate) measurement; P5.3 electrocardiogram (resting 12‐lead electrocardiogram); P5.4 dynamic electrocardiogram (a small box over the shoulder); P5.5 echocardiography; P5.6 smartphone + APP; P5.7 smartwatch/bracelet +APP; P5.8 wearable vest.

Figure 2 shows the practice of the participants towards AF and AF screening. About 74% of the participants were willing to take oral anticoagulants if recommended by their doctors. To prevent the occurrence of AF, approximately 80% of the participants agreed to strictly monitor blood pressure and blood sugar, quit smoking and alcohol consumption, and exercise at an appropriate intensity. The participants showed that they were willing to accept the following AF screening methods if necessary: the top three were blood pressure (heart rate) testing, pulse palpation, and ECG (resting 12‐lead ECG); the bottom three were smartphone combined with the application (app), smart watch/bracelet combined with the app, and wearable vest.

**FIGURE 3:**
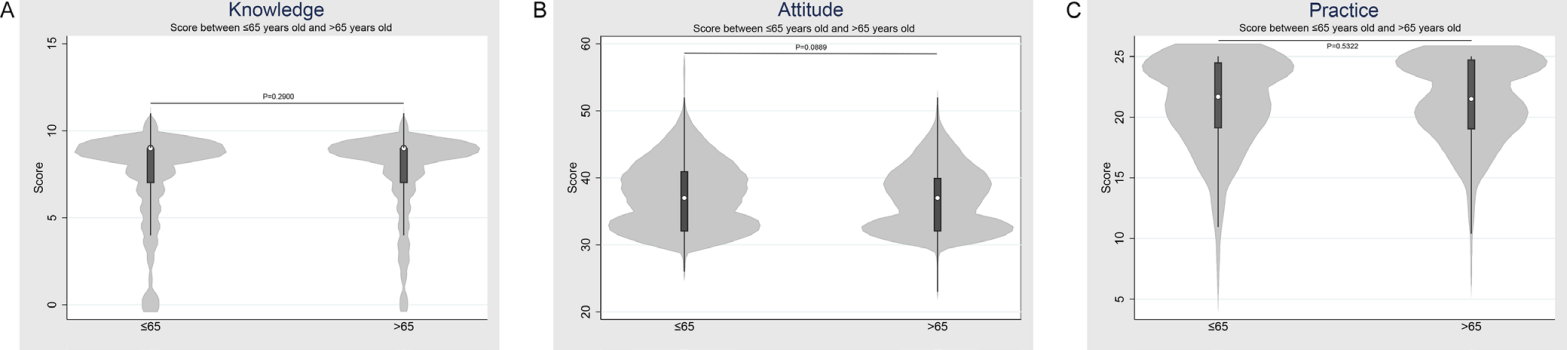
Distribution of KAP scores for participants ≤ 65 versus > 65 years. Distribution of KAP scores for participants ≤ 65 versus > 65 years.

There were no significant differences in knowledge, attitude, and practice between participants aged 65 years or younger and those older than 65 years (Figure 3).

### 3.3 Multivariate Logistic Regression of Practice

The multivariate logistic regression analysis showed that only knowledge (OR = 1.159, 95% confidence interval (CI): 1.045–1.286, *p* = 0.005) was independently associated with better practice, while attitude (OR = 0.861, 95% CI: 0.817–0.908, *p* < 0.001), reported poor sleep quality (OR = 0.529, 95% CI: 0.259–1.0788, *p* = 0.010), fair (OR = 0.234, 95% CI: 0.111–0.491, *p* < 0.001) or fluctuating (OR = 0.321, 95% CI: 0.103–0.996, *p* = 0.049) emotional state, and acquiring the questionnaire through social media (OR = 0.419, 95% CI: 0.268–0.653, *p* < 0.001) were independently associated with worse practice (Table 3).

**TABLE 3:** Univariate and multivariate logistic regression analysis of pratice*.

Variables	Univariate logistic regression		Multivariate logistic regression	
	OR (95% CI)	*p*	OR (95% CI)	*p*
Knowledge score	1.401 (1.270, 1.544)	< 0.001	1.159 (1.045, 1.286)	0.005
Attitude score	0.746 (0.711, 0.782)	< 0.001	0.861 (0.817, 0.908)	< 0.001
Age	0.999 (0.986, 1.012)	0.954		
BMI	1.082 (1.032, 1.135)	0.001	1.056 (0.983, 1.135)	0.131
Sex				
Male	Ref.		Ref.	
Female	0.616 (0.453, 0.837)	0.002	0.687 (0.397, 1.189)	0.180
Residence				
Urban area	Ref.			
Nonurban area	0.731 (0.432, 1.238)	0.245		
Educational level				
Primary school or lower	Ref.			
Junior middle school/senior middle school/technical secondary school	0.963 (0.559, 1.659)	0.893		
Junior college/college	1.219 (0.734, 2.025)	0.443		
Postgraduate or higher	2.031 (0.911, 4.530)	0.083		
Occupation				
Others	Ref.		Ref.	
Professional and technical staff	2.024 (1.412, 2.900)	< 0.001	1.191 (0.715, 1.983)	0.501
Employee	1.424 (0.924, 2.194)	0.109	0.807 (0.441, 1.475)	0.487
Income				
≤ 5000	Ref.		Ref.	
(5001–10,000)	2.622 (1.686, 4.077)	< 0.001	1.049 (0.590, 1.867)	0.869
≥ 10,001	2.410 (1.473, 3.944)	< 0.001	1.144 (0.595, 2.199)	0.686
Marital status				
Married	Ref.		Ref.	
Unmarried	0.466 (0.284, 0.767)	0.003	0.833 (0.427, 1.625)	0.593
Smoking				
Nonsmoker	Ref.		Ref.	
Previous smoker	0.259 (0.132, 0.510)	< 0.001	0.437 (0.175, 1.088)	0.076
Current smoker	0.613 (0.297, 1.266)	0.186	0.604 (0.203, 1.795)	0.365
Alcohol drinking				
Nondrinker	Ref.		Ref.	
Previous drinker	0.451 (0.275, 0.738)	0.002	0.901 (0.436, 1.864)	0.781
Current drinker	0.290 (0.154, 0.544)	< 0.001	0.443 (0.186, 1.052)	0.065
Family history of atrial fibrillation				
No	Ref.		Ref.	
Yes	1.619 (1.077, 2.435)	0.02	1.164 (0.630, 2.152)	0.627
History of atrial fibrillation				
No atrial fibrillation	Ref.			
Previous atrial fibrillation	1.492 (0.989, 2.252)	0.056		
Current atrial fibrillation	0.861 (0.460, 1.608)	0.639		
Unclear	0.603 (0.402, 0.904)	0.015		
Sleep score				
Very good	Ref.		Ref.	
Good	0.076 (0.048, 0.121)	< 0.001	0.313 (0.167, 0.588)	< 0.001
Fair	0.062 (0.039, 0.097)	< 0.001	0.529 (0.259, 1.078)	0.080
Poor/very poor	0.036 (0.018, 0.070)	< 0.001	0.292 (0.115, 0.743)	0.010
Emotional state				
Highly stable	Ref.		Ref.	
Stable	0.089 (0.059, 0.133)	< 0.001	0.370 (0.203, 0.673)	0.001
Fair	0.052 (0.032, 0.086)	< 0.001	0.234 (0.111, 0.491)	< 0.001
Fluctuating	0.064 (0.028, 0.148)	< 0.001	0.321 (0.103, 0.996)	0.049
Obtaining access				
Hospital	Ref.		Ref.	
Social Media	0.152 (0.108, 0.214)	< 0.001	0.419 (0.268, 0.653)	< 0.001

* The 70% of the Practice score, i.e., 24.25, was used as the cutoff for the classification of the Practice score.

## 4 Discussion

Study participants from high‐risk AF population demonstrated acceptable practice towards AF screening and prevention. Findings suggest that better knowledge might result in better practice, while better attitudes do not always influence practice. Additionally, poor sleep and fluctuating emotional states may result in lower practice scores. These findings may help to develop targeted education measures to improve the screening adherence and AF prevention strategies in the high‐risk elderly population.

In this study, 70% of questions regarding the AF prevention and AF screening knowledge were correctly answered, and the total knowledge scores were relatively promising. However, there was insufficient understanding of some issues, such as the relationship between AF and age, the relationship between AF and ischemic stroke or arterial embolism. This was consistent with previous studies, which also demonstrated significant knowledge gaps among patients, caregivers, and physicians in relation to AF and stroke management [18, 19, 20, 21, 22], suggesting that additional health education is needed in this population. It was found that knowledge scores significantly differed according to residence area, educational level, occupation, income, marital status, medical insurance, smoking, alcohol drinking, emotional state, and means of acquiring the questionnaire, outlining the characteristics of the most vulnerable subpopulations in need of educational intervention. In particular, medical resources are concentrated in urban areas; thus, rural residents have fewer possibilities to increase their health‐related knowledge. Those with higher education levels, higher income, higher occupational technology, and better medical security pay more attention to medical information and health knowledge and might better understand AF and AF screening, while others need special guidance. Consistent with the results obtained in the studies on self‐management of AF patients [23, 24, 25], individuals with a lack of educational opportunities and lower income are more vulnerable to the lack of knowledge, which could lead to the lack of understanding towards AF screening as well. Conversely, according to another study, higher income level is not always associated with increased AF knowledge [21], and other factors might play a more notable role.

From the score distribution of the attitude dimension, it was observed that most participants had some awareness about the risk of AF, the importance and advantage of AF screening, and the need for learning more about AF and AF screening. However, some participants believed that the anticoagulation treatment of AF is complex and a long therapy, which may influence their willingness to take AF prevention measures. In another study, Obamiro et al. [26] also reported knowledge/attitude gaps in patients with AF taking oral anticoagulants, making it an important topic for health education. Additionally, some participants were concerned about the cost of AF screening, while others believed that the procedure is time‐consuming and useless, confirming that doubts expressed in other populations [27, 28, 29] are relevant for these study participants as well. Therefore, education regarding screening modalities that reduce cost, save time, and have a reliable detection rate can contribute to improving AF screening practice.

Practice aspects discussed in the present study included willingness to receive oral anticoagulants if prescribed by a doctor, monitoring the blood pressure and blood glucose level, and changing lifestyle habits (moderate physical exercises, quitting smoking and alcohol) to prevent the AF occurrence; in addition, a number of methods were proposed to assess the preference for screening modalities. Similar to the knowledge scores, the lower practice scores were found in the participants who were nonurban residents, lower income group, unmarried, without insurance, current smokers, current drinkers, poor/very poor sleep, and fluctuating emotional state. Many of those categories were also previously reported to have greater AF hospitalization risk [30], stressing the importance of targeted health education. Besides, the results of logistic regression analysis confirmed the association between the participants' knowledge and practice scores, suggesting that targeted interventions to strengthen knowledge could be effective in changing inappropriate behaviors. As recommended by other studies, some interventions can be accomplished at the individual level, such as quitting smoking and drinking, improving sleep quality, and maintaining a relatively stable emotional state [31, 32, 33]. Other interventions should be implemented by the government or medical institutions, including raising the income of residents, improving health insurance, and conducting health education through public welfare activities. Unlike the relationship between knowledge and practice, attitude and practice scores of AF and AF screening were negatively correlated in this study. This may be at least partly attributed to the complex relationship between screening costs and other worries that were expressed in the attitude section, with participants preferring to understand AF screening methods but needing to comply with the hospital requirements [29]. Although Steinhubl et al. [34] reported that immediate monitoring with a home‐based wearable device had a higher rate of AF diagnosis in comparison to delayed monitoring, wearable devices were the least acceptable screening modality in the present study. It is interesting to note that this study accessed a population of older, predominantly female individuals, and the screening methods least acceptable to the population involved newer technology (smartphone applications, wearing smart watch/bracelet, etc.); it is in agreement with the previous study reporting women aged ≥ 50 years less likely to use technology apps for healthcare advice/screening [35]. However, another recent study points out that attitudes of the elderly population towards new screening technologies such as smart watches are not necessarily negative– it is usability and accessibility that concern potential older users [36]. Based on that, future educational interventions should consider barriers for the use of newer technology in the discussed population.

The present study has a few limitations. First, although the findings were valuable for providing targeted improvement measures and interventions for AF prevention and screening, we could not assess and describe the effects of interventions on improving the KAP among the high‐risk population due to the characteristics of the cross sectional design. Moreover, the study's results were mainly based on self‐reported instruments, which may introduce reporting bias and overestimate real results. Although the questionnaire demonstrated high internal consistency, participants who were recruited from social media had lower knowledge/practice but significantly higher attitude scores, which might have affected the total results, and a higher rate of deviations from truth without the direct guidance of researchers is not excluded. Finally, the participants in this study were older than 60 years Thus, the results were not applicable to those at high risk of AF at other ages.

## 5 Conclusions

In summary, although the high‐risk elderly population has acquired partial knowledge of AF prevention and screening, their practice could be improved. Several factors can influence the AF prevention and screening practice, including knowledge and attitude, sleep quality, and emotional state. In addition, an appropriate screening modality is critical to improve AF screening practice. Therefore, targeted strengthening of knowledge education related to AF prevention and screening, establishing a correct attitude towards the topic, and selecting appropriate screening methods could help promote better practices.

## Author Contributions

Z.Y., S.X., and X.Y. carried out the studies, participated in collecting data, and drafted the manuscript. L.Z., L.J., S.R., X.Z., and R.L. performed the statistical analysis and participated in its design. J.Q., C.W., N.G., Y.W., and D.G. participated in the acquisition, analysis, or interpretation of data. All authors read and approved the final manuscript.

## Ethics Statement

This work has been carried out in accordance with the Declaration of Helsinki (2000) of the World Medical Association. The study was approved by the Ethics Committee of the Aerospace Center Hospital (CR‐2021‐CHDRP (2020)‐002). Informed consent was obtained from all the participants.

## Consent

The authors have nothing to report.

## Conflicts of Interest

The authors declare no conflicts of interest.

## Data Availability

All data generated or analyzed during this study are included in this published article and its Supporting Information files.
